# Laparoscopic vs. Open Repair Surgery for the Treatment of Communicating Hydrocele in Children: A Retrospective Study From a Single Center

**DOI:** 10.3389/fsurg.2021.671301

**Published:** 2021-05-12

**Authors:** Jie Liu, Rui Tang, Xiao Wang, Bangzhi Sui, Zhiyuan Jin, Xudong Xu, Qinghua Zhu, Jin Chen, Honglong Ma, Guangqi Duan

**Affiliations:** Department of Pediatric Surgery, Yijishan Hospital of Wannan Medical College, Wannan Medical College, Wuhu, China

**Keywords:** laparoscopy, open surgery, hydrocele, child, minimal invasive

## Abstract

**Purpose:** This study evaluated the outcomes of laparoscopic repair (LR) and open repair (OR) surgery for communicating hydrocele in children.

**Patients and Methods:** We collected the clinical data and follow-up data of all boys (<14 years) who underwent communicating hydrocele surgery in the pediatric surgery department at Yijishan Hospital of Wannan Medical College from January 2017 to December 2018 and retrospectively analyzed the data.

**Results:** In this study, 155 patients were retrospectively enrolled, including 90 patients in the OR group and 65 patients in the LR group. There were significant differences in operation time and the recurrence of hydrocele between the two groups. The persistence of scrotal swelling in the LR group was significantly lower than that in the OR group. There was no significant difference in postoperative hospitalization time or incision infection rate between the two groups.

**Conclusion:** In conclusion, this study shows that laparoscopic treatment of children with communicating hydrocele has the advantages of a hidden incision, a shortened operation time, and a reduced postoperative recurrence rate and can be used as the preferred surgical method. However, laparoscopic treatment should be selected according to the specific condition of each child and cannot completely replace traditional open surgery.

## Introduction

Hydrocele in children is one of the common congenital diseases in boys (1, 2). The main reason for this is that intraperitoneal fluid enters the scrotum through a congenital patent processus vaginalis (PPV), thus forming hydrocele (3, 4). Children with a communicating hydrocele are different from adults, and simple high ligation of the PPV can achieve satisfactory results (3, 5). Traditional open repair (OR) surgery has the characteristics of a simple operation and strong popularity and it is accepted by the majority of pediatric surgeons. However, some scholars believe that OR leads to considerable postoperative pain, can cause damage to the spermatic cord blood vessels and vas deferens, leaves a visible postoperative incision, and has other problems (6, 7). Laparoscopic repair (LR) of the PPV in the treatment of hydrocele in children has the characteristics of hidden scarring, less trauma, and less postoperative scrotal swelling. Proponents believe that laparoscopic treatment of hydrocele may gradually replace open treatment of hydrocele in children (6–8). However, there is still controversy as to whether laparoscopic treatment of children with hydrocele has significant advantages (7, 9). In this study, we collected and retrospectively analyzed the clinical and surgical data of laparoscopic treatment of children with communicating hydrocele in our center and compared the advantages and disadvantages of the two surgical methods (OR and LR groups) to provide a reference for the clinical treatment of children with communicating hydrocele.

## Materials and Methods

We retrospectively analyzed the clinical data of all male children (<14 years old) who underwent hydrocele repair in the pediatric surgery department of Yijishan Hospital of Wannan Medical College from January 2017 to December 2018. All of the methods were approved by the ethics committee of Yijishan Hospital of Wannan Medical College, and written consent was obtained from the children's parents. We paid attention to personal privacy and confidentiality when collecting the data and did not disclose the identity of the child to anyone. In this study, our senior surgeons explained the causes of hydrocele in children to their parents and told them that there were two kinds of operation methods (OR and LR) and that the parents were free to choose the specific type of operation.

### Data Collection

The clinical data were collected by consulting the medical records of the children. The clinical data included the age of the child at admission, the location of the hydrocele, the final type of operation, the laterality of the hydrocele diagnosed before and after the operation, operation time, postoperative hospitalization time, and postoperative complications.

### Surgical Methods

**Figure 1:**
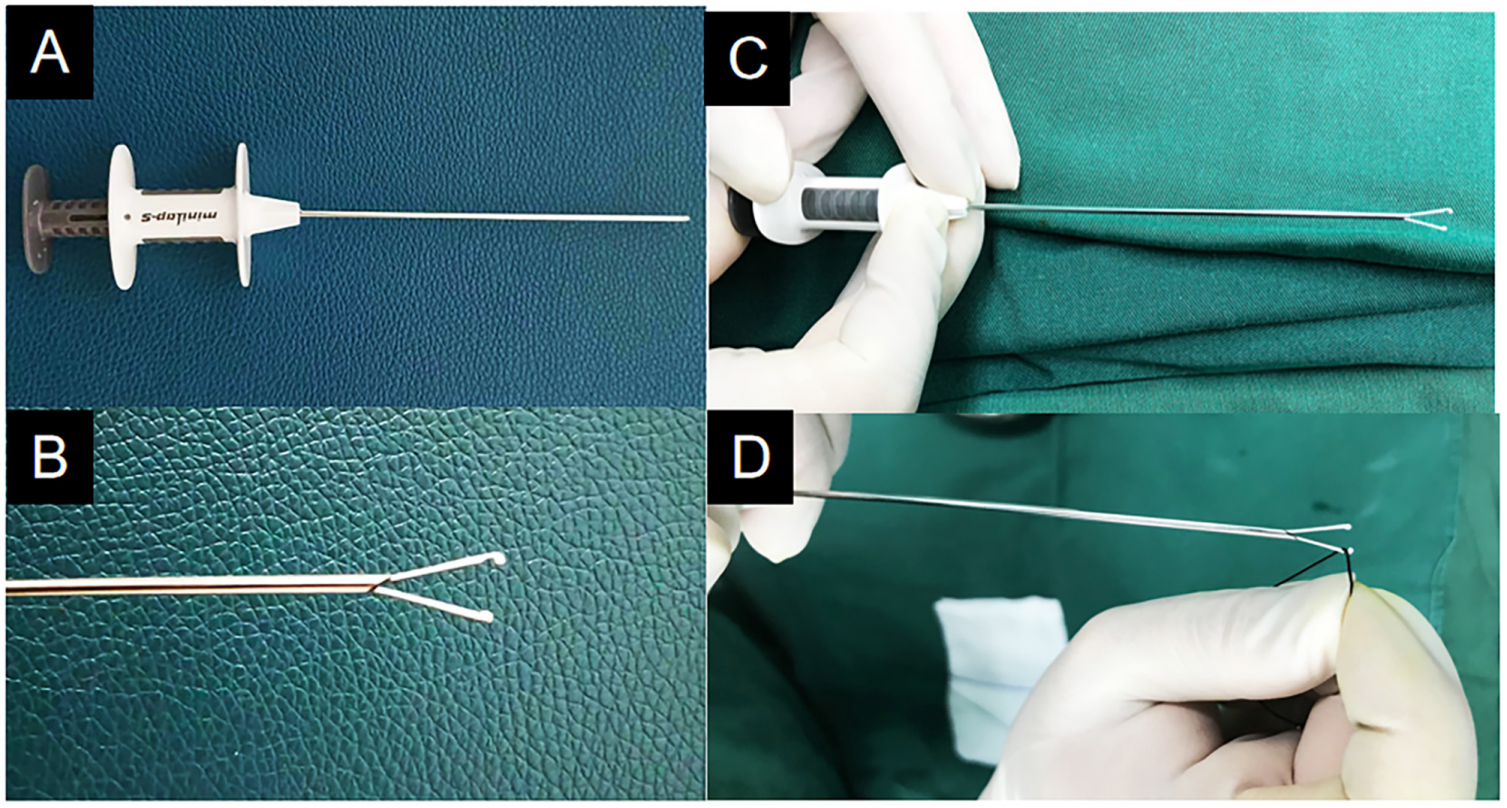
The abdominal wall suture device with water injection function. **(A)** The appearance image. **(B)** The front end of the injection needle core is two retractable concave arc-shaped metal hooks. **(C)** Pressing the end of the abdominal wall suture device can open the claw of the needle. **(D)** A spring is installed in the sheath of the hand-held part of the back end, which is convenient for the operator to pull out the needle core and hook the ligation line at the front end of the needle core, and then it can automatically spring back to insert and fasten.

In the LR group, laparoscopic percutaneous extraperitoneal closure (LPEC) was performed extraperitoneally with an abdominal wall suture device with a water injection function (Figure 1), which was produced by Xiamen Surgaid Medical Equipment Co., Ltd., of China (Patent No.: ZL 201620804971.6). The suture device was a double trocar, 15 cm in length. The diameter of the outer sheath trocar was 1.5 mm, and the diameter of the inner trocar was 1 mm. The front end of the injection needle core has two retractable concave arc-shaped metal hooks. The two hooks are pushed forward and gradually opened to hook the ligation line into the abdominal cavity. The two claws are retracted backward and gradually closed to facilitate hanging of the abdominal ligation line. A spring is installed in the sheath of the hand-held part of the back end, which is convenient for the operator to pull out the needle core and hook the ligation line at the front end of the needle core, and then it can automatically spring back to insert and fasten. The tail end is butterfly-shaped and easy to handle (Figure 1). In the design, the 1/3 inclined plane of the front end of the obturator is curved into an arc, which is convenient for wire winding and ligation. A 30° laparoscope with a built-in 5.5 mm aperture (Storz, Germany) was used.

All patients underwent general anesthesia, urination before the operation and supine positioning after anesthesia. To perform the procedure: make an arc-shaped incision of the umbilicus or the lower edge of the umbilicus, place the trocar with a 5 mm thread and a 30° laparoscope, and observe the closure of the bilateral processus vaginalis after routine exploration of the internal organs in the abdominal cavity. Under laparoscopic monitoring, the skin dermis is punctured with the tip of the knife at the position corresponding to the lower abdominal transverse lines of the processus vaginalis of the hydrocele. A 2-0 silk thread is taken. One end of the thread is hooked on the suture device and stretched out of the two arc-shaped metal hooks. The ligation line and the ligation needle were parallel with the ligation needle outside. At the body surface marking point, the needle is punctured through the muscle layer of the abdominal wall to reach the extraperitoneal space of the anterior wall of the processus vaginalis. Before crossing the vas deferens, the modified crochet inclined plane is deviated from the operator's visual field against the vas deferens, and the retroperitoneum is jacked up (if it is difficult to pass, normal saline is injected into the tail to facilitate separation).

**Figure 2:**
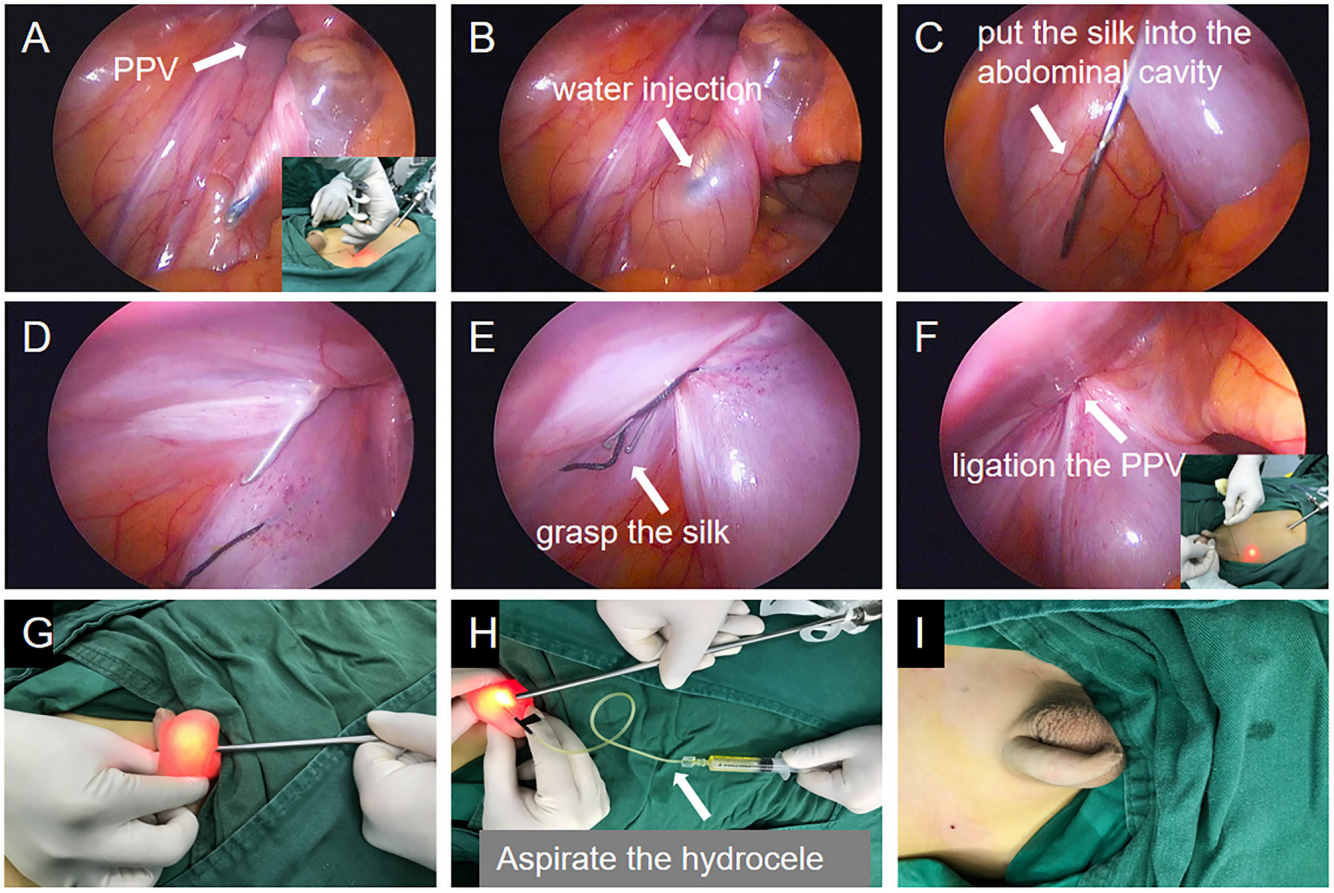
The images of single-port laparoscopic percutaneous extraperitoneal closure operation steps with the abdominal wall suture device with water injection function. **(A)** Crochet with thread was inserted into the lateral inferior epigastric artery. **(B)** The hook needle with thread in the extraperitoneal space sneaked along the inner side of the processus vaginalis to the vas deferens. The inclined plane of the crochet deviated from the operator's field of vision, against the vas deferens, and injected normal saline to jack up the retroperitoneum and cross the space between the vas deferens and the retroperitoneum. **(C)** After crossing the spermatic vessels, the peritoneum was punctured and the silk thread was placed in the abdominal cavity. **(D)** The crochet needle sneaked along the outer side of the inner ring in the extraperitoneal space. **(E)** Hook the silk thread in the abdominal cavity. **(F)** Knot in the extraperitoneal space and close the processus vaginalis. **(G)** Laparoscopic lens is helpful to identify hydrocele. **(H)** The liquid in the tunica vaginalis was aspirated by syringe. **(I)** The photograph of the scrotum after the operation.

With the help of the crochet spade, the space between the vas deferens and the retroperitoneal cavity is pushed forward to reach the danger triangle (known as the triangle of doom; formed by the spermatic vessels and vas deferens, which meet at the vaginal process and enter the inguinal canal). There are external iliac arteries and veins in the triangle. The operation should avoid injury to this area, which can cause massive bleeding, which is why clinically, it is known as the triangle of doom. The peritoneum is punctured under the laparoscope, and the silk thread is thrown into the abdominal cavity. After that, the crochet is slowly withdrawn to the outer peritoneum of the anterior wall of the processus vaginalis, and then the trocar needle is sneaked into the retroperitoneal space outside the spermatic vessels along the lateral extraperitoneal space of the processus vaginalis mouth. The crochet slope is deviated from the operator's field of vision, butted against the spermatic vessels, and pushed forward. The occluder extends the metal hook to hang the preset thread end, retracted and clamped, pulling the silk thread at both ends. Then, remove the ligation line from the body, ligate and close the PPV, and bury the suture knot under the abdominal wall muscle layer and outside the peritoneum of the anterior wall of the processus vaginalis. If there is a PPV on the opposite side, the same method should also be applied for ligation. The pneumoperitoneum machine is stopped, the laparoscopic lens is withdrawn from the abdominal cavity, and the scrotal effusion is extracted with a syringe assisted by the lens. There is no need to close the puncture hole of the abdominal transverse striation ligation needle; only the incision of the umbilical fascia is sutured, and the skin edge tissue is glued (Figure 2).

In the OR group, the transverse incision is made ~1.5 cm along the dermatoglyphic line in the affected groin, and the sheath process is separated and found. Then, high ligation of the sheath process is performed with silk thread ligation at the PPV, part of the tunica vaginalis is removed, or the tunica vaginalis is reversed. The vas deferens should be protected from damage when the tunica vaginalis is removed.

### Follow-Up Schedule

The follow-up of this study was completed by outpatient follow-up after discharge and telephone follow-up in our hospital follow-up center, but telephone follow-up was only to make an appointment for the specific time of outpatient follow-up. If the outpatient pediatrician found that the scrotum was larger than the healthy side, all patients received a scrotum B-ultrasound examination. The persistence of scrotal swelling after the operation refers to the findings on a scrotal B-ultrasound examination 6 months after the operation showing that the scrotum is still larger than that of the healthy side, but there is no recurrence of hydrocele. Patients were followed up until December 2020.

### Statistical Analysis

All statistical analyses in this study were carried out using the R programming language, version 3.6.0 (R Foundation), in which a *P* < 0.05 was considered statistically significant.

## Results

From January 2017 to December 2018, a total of 155 boys in our center were retrospectively enrolled in this study, including 90 patients in the OR group and 65 patients in the LR group. The hydrocele operation was successfully completed for all patients. All of our patients received outpatient follow-up from 12 to 24 months, with an average of 18.6 months. The comparison of preoperative clinical characteristics between the two groups is shown in Table 1, showing no significant differences in preoperative baseline characteristics between the two groups.

**Table 1:** Demographic data of the OR and LR groups before operation.

**Characteristics**	**OR**	**LR**	***t* (*x*^**2**^) value**	***P*-value**
Median age (months)	41 (12~90)	43 (5~116)	*t* = 0.379	0.705
Laterality (preoperative, No.)			*x*^2^ = 0.484	0.487
Unilateral	87	64		
Bilateral	3	1		

*OR, open repair; LR, laparoscopic repair; No., number*.

**Figure 3:**
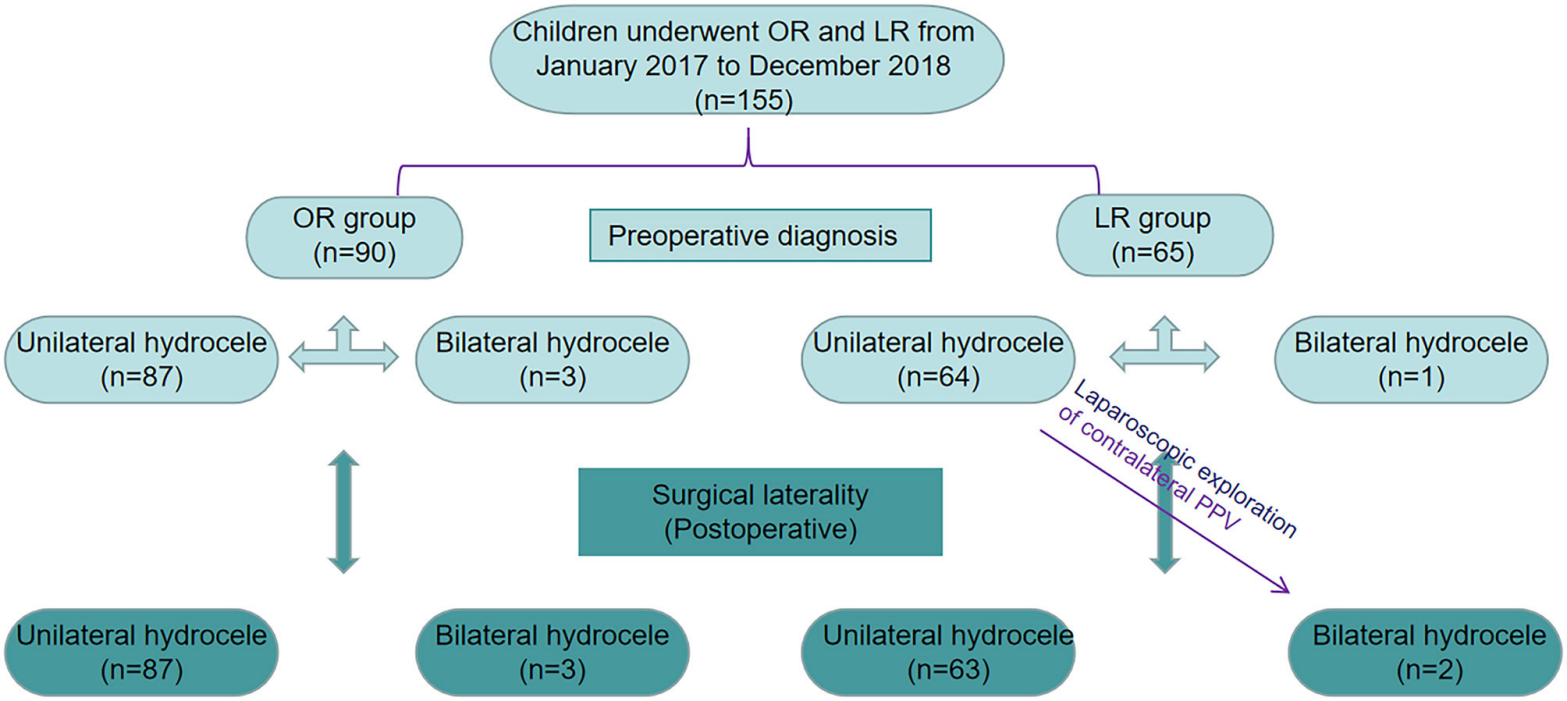
Identification of patients underwent open surgery communicating hydrocele repair and laparoscopic hydrocele repair.

For the differences in postoperative diagnosis, operation time, and postoperative hospitalization time between the two groups, the results showed that 89 patients in the OR group underwent unilateral hydrocele surgery and 1 patient underwent bilateral surgery. In the LR group, 63 patients underwent unilateral hydrocele surgery and 2 patients underwent bilateral surgery. There was no significant difference in lateral position between the two groups (*P* = 0.929). In the LR group, only one of 64 children with unilateral hydrocele had a contralateral PPV (1.6%). Ligation was performed at the same time in bilateral cases (Figure 3).

The mean operation time was 50.37 ± 9.72 min in the OR group and 18.50 ± 6.15 min in the LR group. The operation time in the OR group was significantly longer than that of the LR group (*P* < 0.001; Table 2). The mean postoperative hospitalization time was 1.222 ± 0.055 days in the OR group and 1.167 ± 0.043 days in the LR group, a difference that was not significant (*P* = 0.325; Table 2).

**Table 2:** Comparison of operation site, operation time, and postoperative hospitalization time between the two groups.

**Characteristics**	**OR**	**LR**	***t* (*x*^**2**^) value**	***P-*value**
Operation time (min)	50.37 ± 9.72	18.50 ± 6.15	*t* = 33.13	<0.001
Laterality (preoperative, No.)			*x*^2^ = 0.008	0.929
Unilateral	87	63		
Bilateral	3	2		
Postoperative hospitalization days	1.222 ± 0.055	1.167 ± 0.043	*t* = 0.987	0.325

*OR, open repair; LR, laparoscopic repair*.

To compare the postoperative effects of the two surgical methods, we analyzed the recurrence rate and postoperative complications of the two groups. The results are shown in Table 3. In this study, six cases of ipsilateral hydrocele recurred in the OR group (6.67%), and no recurrence occurred in the LR group. The recurrence rate in the OR group was significantly higher than that in the LR group (*P* = 0.034). Incision infection occurred in three patients in the OR group (3.33%) and in one patient in the LR group (1.54%), a difference that was not significant (*P* = 0.487). All of the infected incisions were effectively controlled and healed after dressing changes. The persistence of scrotal swelling in the OR group (10%) was significantly higher than that in the LR group (1.54%) (*P* = 0.034). There were two cases of iatrogenic testicular upward movement in the OR group and none in the LR group, but there was no significant difference between the two groups (*P* = 0.226). In this study, there was no case of testicular atrophy after surgery in any patients with hydrocele.

**Table 3:** Postoperative complications in the OR and LR groups.

**Complications**	**OR**	**LR**	***x*^**2**^**	***P-*value**
Ipsilateral recurrent hydrocele	6/90 (6.67%)	0/65	4.508	0.034
Surgical site infection	3/90 (3.33%)	1/65 (1.54%)	0.484	0.487
Scrotal swelling	9/90 (10%)	1/65(1.54%)	4.477	0.034
Iatrogenic ascent of the testis	2/90 (2.22%)	0/65	1.463	0.226
Testicular atrophy	0/90	0/65	–	–

*OR, open repair; LR, laparoscopic repair*.

## Discussion

Hydrocele in children is a common disease addressed in pediatric surgery and it is related to congenital PPV (10, 11). In the embryonic stage, the processus vaginalis begins to appear at 12 weeks of gestation. When the processus vaginalis herniates out of the abdominal wall and passes through the inguinal canal, the protruding muscular layer finally forms the scrotal adventitia (12). In the inguinal canal and the adjacent sheath process, the testicular girdle develops into a thick stromal structure, extends to the head side, enters the peritoneum, and faces the tail end of the testis, and matures at a position equivalent to the inner ring. Then, the testis completes the descent process into the inguinal canal and scrotum under the action of various factors (12). After the testis enters the scrotum, the processus vaginalis narrows in the middle part of the spermatic cord and becomes tubular. Finally, it is closed above the testis, leaving part of the vagina around the testis, which becomes the tunica vaginalis (11, 13). In this development process, if the processus vaginalis is not closed completely and the volume of fluid in the tunica vaginalis cavity is too large, hydrocele can occur. An operation to close the PPV is the preferred treatment (13, 14).

The traditional OR surgery method first needs to open the inguinal canal and find the PPV for high ligation and turnover of the tunica vaginalis (15). The evolution of OR surgery from the early oblique inguinal incision to the later transverse incision or scrotal incision is mainly concerned about how to reduce intraoperative trauma and achieve a better cosmetic effect (9, 15). However, in an actual operation, the PPV is located in the inner front of the spermatic cord, which is a transparent membrane. The lumen of the sheath process tube in children with hydrocele is usually thin, and there is a tendril-like venous plexus in the outer front of the spermatic cord, including the vas deferens and arteries, and the cremaster around it. Because the vaginal process tube is small, sometimes it is difficult to find during the operation, so it is easy to accidentally damage the spermatic cord blood vessels, vas deferens, cremaster, etc. After the operation, local bleeding, swelling, and scar adhesion often appear, and severe cases can exhibit testicular atrophy (16). In this study, the rate of scrotal swelling in the OR group 6 months after the operation was 10% due to partial excision of the tunica vaginalis or tunica vaginalis turnover. Additionally, we also realized that when the adhesion around the PPV was obvious, it was difficult for the operator to dissociate it to a higher position, which caused inaccurate ligation or at a low position. In addition, the wall of the PPV is thin; if the separation force is too large, it easily tears. All of the above are risk factors for postoperative recurrence (16).

In recent years, with the development and popularization of laparoscopic technology, many medical centers have carried out laparoscopic surgery in the treatment of children with communicating hydrocele (17, 18). Wang et al. (17) used laparoscopic techniques for the treatment of hydrocele in children. They placed the laparoscope and needle holding forceps into the umbilicus. At the same time, a suture needle with thread was inserted into the abdominal cavity from the abdominal wall. The needle holding forceps were used to purse-string suture the PPV, and a single instrument was used to tie the knot and ligate the PPV. In recent years, a series of improved LPEC techniques have been reported, and the surgical instruments are different (19, 20). Liu et al. (8) used a modified Kirschner pin for single-port LPEC in the treatment of 81 cases of children with hydrocele, and all patients achieved good results. In addition, other surgeons have also reported that homemade hernia needles, epidural puncture needles, syringe needles, and other suture instruments have achieved good therapeutic effects (6, 16, 19–21).

In addition, with the development of laparoscopic technology, surgeons have also carried out a large number of comparative studies on OR and LR in the treatment of children with hydrocele (22–24). Zhang et al. (13) reported a retrospective study of 1,332 boys with hydrocele in two centers, including 382 cases in the OR group and 950 cases in the LR group. The LR group was divided into three groups: conventional double port, transumbilical single site double port, and transumbilical single port. The authors believe that LR has more advantages than traditional OR in reducing the operation and hospitalization time and reducing the incision size. At the same time, the authors consider that a transumbilical single site double hole operation is the best way to treat hydrocele in boys. Miyake et al. (22) reported a retrospective study of 1,050 cases of OR and 1017 cases of LR in the treatment of children with hydrocele. The authors believe that LR can reduce the operation time and simultaneously deal with a contralateral metachronous hernia, but there was no significant difference in the recurrence rate of hydrocele between the two groups. However, some surgeons have different views that open surgery has also achieved good surgical results (1, 18, 25). In this study, we used an abdominal wall suture device with the function of water injection for LPEC for the treatment of communicating hydrocele in boys. To the best of our knowledge, this is the first study on this device.

Through retrospective analysis of the clinical data of two different surgical methods, we believe that LR for children with communicating hydrocele has the following advantages compared with OR: (1) compared with OR, LR can ensure high ligation of the PPV, which can reduce the postoperative recurrence rate (2, 26) the abdominal wall suture device with water injection function used in single-port LPEC only needs one puncture to complete the extraperitoneal ligation, which avoids the possibility of ligating muscles or other tissues, and reduces the postoperative recurrence rate; (3) the laparoscopic lens has the function of magnifying. The operator can clearly distinguish the spermatic vein and vas deferens in the abdominal cavity to avoid damage to the surrounding tissue; (4) LPEC does not need to dissect the inguinal canal, which greatly reduces the operation time. Even if the suture needle punctures the peritoneum in many places, the PPV can be completely ligated and closed. This is more advantageous for patients and reduces damage to the surrounding tissues; (5) the laparoscopic operation is simple, and the learning curve is short and easy to master. For junior doctors, it can become an entry-level operation to learn laparoscopic surgery technology; (6) the wound after the laparoscopic surgery is beautiful and hidden, basically scarless surgery, which fully embodies the concept of minimally invasive surgery and this is appreciated by the parents of the children. Interestingly, one of the most important characteristics of laparoscopy is that it can detect the contralateral PPV. However, in our study, only one of 64 patients with unilateral hydrocele in the LR group had contralateral PPV, which is far lower than in other reports. We think this may be related to our cases all being treated at a single center. We need a larger sample to further analyze the specific reasons for this discrepancy.

Although laparoscopic surgery has achieved good results in the treatment of children with communicating hydrocele, everything has two sides. Compared with open surgery, we think that laparoscopic surgery still has some problems. First, improper operation of trocar puncture in laparoscopic surgery may damage the intestinal tube or cause subcutaneous emphysema. Second, LR requires tracheal intubation or laryngeal mask-assisted general anesthesia, which may lead to an increase in the incidence of postoperative upper respiratory tract infection. Third, due to the existence of cardiopulmonary disease in some children before surgery, based on safety considerations, the anesthesiologist may recommend OR as the first choice to avoid interference from the pneumoperitoneum on the airway pressure of the child during surgery.

As a preliminary study, we also recognize several potential weaknesses in this study. For example, all cases in this study were from a single center, which may have had some impact on our analysis results. There was selection bias, as the parents were allowed to choose the type of surgery, and the surgeons' biases in presenting the two options could have influenced parental decision making. The difference in outcomes could be influenced by the number of surgeons performing the surgery in each of the two cohorts and their relative expertise in performing each type of procedure. In addition, the postoperative follow-up time has not yet reached puberty, so there is no comparison of the impact of the different surgical methods on the fertility of the patients.

## Conclusions

In conclusion, this study showed that laparoscopic treatment of children with communicating hydrocele has the advantages of a hidden incision, a shortened operation time, and a reduced postoperative recurrence rate and can be used as the preferred surgical method, but it should be selected according to the specific condition of each child and cannot completely replace traditional open surgery.

## Data Availability Statement

The original contributions generated for the study are included in the article/Supplementary Material, further inquiries can be directed to the corresponding author/s.

## Ethics Statement

The studies involving human participants were reviewed and approved by ethics committee of Yijishan Hospital of Wannan Medical College. Written informed consent to participate in this study was provided by the participants' legal guardian/next of kin.

## Author Contributions

JL and GD designed the research. JL, RT, and XW obtained the clinical data and analysis of the results. XX, BS, and ZJ prepared the tables. QZ, JC, and HM prepared the figures. JL and GD revised the manuscript. All authors have approved the final version of the manuscript.

## Conflict of Interest

The authors declare that the research was conducted in the absence of any commercial or financial relationships that could be construed as a potential conflict of interest.

## Abbreviations

PPV: Patent processus vaginalis
OR: Open repair
LR: Laparoscopic repair
LPEC: laparoscopic percutaneous extraperitioneal closure.

## References

[1] Oh JH Chung HS Yu HS Kang TW Kwon D Kim SO. Hydrocelectomy via scrotal incision is a valuable alternative to the traditional inguinal approach for hydrocele treatment in boys. Investig Clin Urol. (2018) 59:416–21. 10.4111/icu.2018.59.6.416 30402575 PMC6215779

[2] Jobson M Hall NJ. Current practice regarding timing of patent processus vaginalis ligation for idiopathic hydrocele in young boys: a survey of UK surgeons. Pediatr Surg Int. (2017) 33:677–81. 10.1007/s00383-017-4085-4 28424863 PMC5434128

[3] Jin Z Wang F. Effectiveness of laparoscopy in the treatment of pediatric hydrocele: a systematic review. J Laparoendosc Adv Surg Tech A. (2018) 28:1531–9. 10.1089/lap.2018.0095 30063415

[4] Cimador M Castagnetti M De Grazia E. Management of hydrocele in adolescent patients. Nat Rev Urol. (2010) 7:379–85. 10.1038/nrurol.2010.80 20548330

[5] Liu J Wu X Xiu W Hao X Zhao J Wei B. A comparative study examining laparoscopic and open inguinal hernia repair in children: a retrospective study from a single center in China. BMC Surg. (2020) 20:244. 10.1186/s12893-020-00912-7 33076895 PMC7574473

[6] Baradaran N Wood CM McCoy OO Prasad MM Stec AA. Laparoscopic intra-abdominal patent processus vaginalis ligation in pediatric urology practice. J Pediatr Urol. (2017) 13:512.e1–e6. 10.1016/j.jpurol.2017.03.026 28465160

[7] Esposito C Escolino M Turra F Roberti A Cerulo M Farina A. Current concepts in the management of inguinal hernia and hydrocele in pediatric patients in laparoscopic era. Semin Pediatr Surg. (2016) 25:232–40. 10.1053/j.sempedsurg.2016.05.006 27521714

[8] Liu W Wu R Du G. Single-port laparoscopic extraperitoneal repair of pediatric inguinal hernias and hydroceles by using modified Kirschner pin: a novel technique. Hernia. (2014) 18:345–9. 10.1007/s10029-013-1181-9 24218078

[9] Alp BF Irkilata HC Kibar Y Zorba U Sancaktutar AA Kaya E. Comparison of the inguinal and scrotal approaches for the treatment of communicating hydrocele in children. Kaohsiung J Med Sci. (2014) 30:200–5. 10.1016/j.kjms.2013.11.006 24656161 PMC11916440

[10] Peng Y Li C Lin W Xu L. Application of a laparoscopic, single-port, double-needle technique for pediatric hydroceles with multiple peritoneal folds: a trial from a single-center 5-year experience. Urology. (2015) 85:1466–70. 10.1016/j.urology.2015.01.053 26099890

[11] Verma S Agrawal V Acharya H Sharma D. Laparoscopic suture-less herniotomy using tissue-sealing device for paediatric hydrocele. J Minim Access Surg. (2019) 16:111–4. 10.4103/jmas.JMAS_251_18 30618434 PMC7176016

[12] Dagur G Gandhi J Suh Y Weissbart S Sheynkin YR Smith NL. Classifying hydroceles of the pelvis and groin: an overview of etiology, secondary complications, evaluation, and management. Curr Urol. (2017) 10:1–14. 10.1159/000447145 28559772 PMC5436019

[13] Zhang Y Chao M Zhang X Wang Z Fan D Zhang K. Does the laparoscopic treatment of paediatric hydroceles represent a better alternative to the traditional open repair technique? A retrospective study of 1332 surgeries performed at two centres in China. Hernia. (2018) 22:661–9. 10.1007/s10029-017-1715-7 29243214 PMC6061066

[14] Palmer LS. Hernias and hydroceles. Pediatr Rev. (2013) 34:457–64; quiz 464. 10.1542/pir.34-10-457 24085793

[15] Chen Y Wang F Zhong H Zhao J Li Y Shi Z. A systematic review and meta-analysis concerning single-site laparoscopic percutaneous extraperitoneal closure for pediatric inguinal hernia and hydrocele. Surg Endosc. (2017) 31:4888–901. 10.1007/s00464-017-5491-3 28389795

[16] Wang F Zhong H Chen Y Zhao J Li Y Chen J. Single-site laparoscopic percutaneous extraperitoneal closure of the internal ring using an epidural and spinal needle: excellent results in 1464 children with inguinal hernia/hydrocele. Surg Endosc. (2017) 31:2932–8. 10.1007/s00464-016-5309-8 27815740

[17] Wang F Shou T Zhong H. Is two-port laparoendoscopic single-site surgery (T-LESS) feasible for pediatric hydroceles? Single-center experience with the initial 59 cases. J Pediatr Urol. (2018) 14:67 e61–7.e66. 10.1016/j.jpurol.2017.09.016 29108870

[18] Choi BS Byun GY Hwang SB Koo BH Lee SR. A comparison between totally laparoscopic hydrocelectomy and scrotal incision hydrocelectomy with laparoscopic high ligation for pediatric cord hydrocele. Surg Endosc. (2017) 31:5159–65. 10.1007/s00464-017-5582-1 28493163

[19] Wang Z Xu L Chen Z Yao C Su Z. Modified single-port minilaparoscopic extraperitoneal repair for pediatric hydrocele: a single-center experience with 279 surgeries. World J Urol. (2014) 32:1613–8. 10.1007/s00345-014-1259-8 24522790

[20] Wang DJ Qiu JG Fang YQ Si TJ Luo JB Gao X. Laparoscopic extraperitoneal repair of symptomatic hydrocele in children: a single-center experience with 73 surgeries. J Endourol. (2011) 25:1221–5. 10.1089/end.2010.0594 21711131

[21] Li S Liu X Wong KKY Liu L Li Y. Single-port laparoscopic herniorrhaphy using a two-hooked cannula device with hydrodissection. J Pediatr Surg. (2018) 53:2507–10. 10.1016/j.jpedsurg.2018.08.009 30227994

[22] Miyake H Fukumoto K Yamoto M Nouso H Kaneshiro M Nakajima H. Comparison of percutaneous extraperitoneal closure (LPEC) and open repair for pediatric inguinal hernia: experience of a single institution with over 1000 cases. Surg Endosc. (2016) 30:1466–72. 10.1007/s00464-015-4354-z 26139500

[23] Saka R Okuyama H Sasaki T Nose S Yoneyama C. Safety and efficacy of laparoscopic percutaneous extraperitoneal closure for inguinal hernias and hydroceles in children: a comparison with traditional open repair. J Laparoendosc Adv Surg Tech A. (2014) 24:55–8. 10.1089/lap.2013.0109 24180356

[24] Saber A. Minimally access versus conventional hydrocelectomy: a randomized trial. Int Braz J Urol. (2015) 41:750–6. 10.1590/S1677-5538.IBJU.2014.0248 26401869 PMC4757005

[25] Matcovici M Tareen F O'Connor BR Gillick J. Adolescent *de novo* hydroceles - should they be dealt with by inguinal or scrotal approach? J Pediatr Surg. (2018) 53:2228–30. 10.1016/j.jpedsurg.2018.08.007 30231973

[26] Huerta S Timmerman C Argo M Favela J Pham T Kukreja S. Open, laparoscopic, and robotic inguinal hernia repair: outcomes and predictors of complications. J Surg Res. (2019) 241:119–27. 10.1016/j.jss.2019.03.046 31022677

